# Effect of the Knee and Hip Angles on Knee Extensor Torque: Neural, Architectural, and Mechanical Considerations

**DOI:** 10.3389/fphys.2021.789867

**Published:** 2022-01-04

**Authors:** Yoann M. Garnier, Romuald Lepers, Patrizio Canepa, Alain Martin, Christos Paizis

**Affiliations:** ^1^INSERM 1093-CAPS, Université Bourgogne Franche-Comté, UFR des Sciences du Sport, Dijon, France; ^2^Université Clermont Auvergne, UPR 3533, Laboratoire AME2P, Clermont-Ferrand, France; ^3^Department of Experimental Medicine, Section of Human Physiology, University of Genoa, Genoa, Italy; ^4^Department of Neuroscience, Rehabilitation, Ophthalmology, Genetics and Maternal Child Health, University of Genoa, Genoa, Italy; ^5^Centre for Performance Expertise, Faculté des Sciences du Sport, Université de Bourgogne, Dijon, France

**Keywords:** maximal voluntary contraction, knee extensors, corticospinal excitability, pennation angle, fascicle length

## Abstract

This study examined the influence of knee extensors’ hip and knee angle on force production capacity and their neuromuscular and architectural consequences. Sixteen healthy men performed sub-maximal and maximal voluntary isometric contractions (MVIC) of knee extensors with four different combinations of the knee and hip angles. Muscle architecture, excitation-contraction coupling process, muscular activity, and corticospinal excitability were evaluated on the vastus lateralis (VL) and rectus femoris (RF) muscles. MVIC and evoked peak twitch (Pt) torques of knee extensors increased significantly (*p* < 0.05) by 42 ± 12% and 47 ± 16% on average, respectively, under knee flexed positions (110° flexion, 0° = full extension) compared to knee extended positions (20° flexion) but were not different between hip positions (i.e., 0° or 60° flexion). Knee flexion also affected VL and RF muscle and fascicle lengths toward greater length than under knee extended position, while pennation angle decreased for both muscles with knee flexion. Pennation angles of the VL muscle were also lower under extended hip positions. Alternatively, no change in maximal muscle activation or corticospinal activity occurred for the VL and RF muscles across the different positions. Altogether these findings evidenced that MVIC torque of knee extensors depended particularly upon peripheral contractile elements, such as VL and RF muscle and fascicle lengths, but was unaffected by central factors (i.e., muscle activation). Furthermore, the hip position can affect the pennation angle of the VL, while VL muscle length can affect the pennation angle of the RF muscle. These elements suggest that the VL and RF muscles exert a mutual influence on their architecture, probably related to the rectus-vastus aponeurosis.

## Introduction

Consistent findings demonstrate the influence that the knee joint exerts angle on the knee extensors’ maximal torque production capacity. Lower maximal voluntary isometric contraction (MVIC) torque was, for instance, developed at a moderate flexed knee position (20–35° knee flexion, 0° = full extension) compared to more flexed positions (55–75° knee flexion; Babault et al., 2003; Cavalcante et al., 2021). The knee extensors encompass the three mono-articular vastii muscles [vastus lateralis (VL), vastus intermedius (VI), and vastus medialis (VM)] and the bi-articular rectus femoris (RF) muscle who share a common termination on the quadriceps tendon. Because the RF muscle originates from the acetabulum, a change in hip joint position could therefore also influence the knee joint’s torque due to the bi-articular RF muscle’s contribution (Herzog and ter Keurs, 1988). This model was further validated by the greater MVIC torque developed in a seated compared to a supine position (i.e., flexed vs. extended hip position; Maffiuletti and Lepers, 2003; Rochette et al., 2003; Ema et al., 2017), while others did not find a difference between these two positions (Bampouras et al., 2017; Cavalcante et al., 2021). Contrary to the general agreement about the influence of knee joint angle on knee extensor’s MVIC, the effect of hip joint angle remains discussed.

Deficits in MVIC torque could result from an alteration in contractile properties of the knee extensor muscles. Previous studies tested the influence of hip or knee joint angle configuration on the excitation-contraction coupling process of the knee extensor using percutaneous electrical stimulation. Similar decrements in MVIC and twitch torque amplitude (Babault et al., 2003) or maximal electrically induced contraction torque (Cavalcante et al., 2021) occurred in moderate knee flexed positions (20°–35° knee flexion) compared to more flexed positions (55–75°). Additionally, Cavalcante et al. (2021) reported that hip position does not influence maximal electrically induced contraction torque irrespective of knee angle so does for the MVIC. These findings led these authors to suggest that the lower MVIC torque would likely result from a mechanical disadvantage impairing knee extensors’ contractile properties (Babault et al., 2003; Cavalcante et al., 2021). However, this hypothesis is questioned by the greater electrically evoked responses elicited in the knee extensors in a supine compared to a seated position despite a lower (Maffiuletti and Lepers, 2003) or a similar (Bampouras et al., 2017) MVIC torque, respectively.

Using ultrasonography, Cavalcante et al. (2021) measured shorter fascicle length and greater pennation angle of the vastii and RF muscles for a knee extended position than a more flexed position, irrespective of hip angle. On the contrary, an increase in hip extension decreased pennation angle and increased the fascicle length of the RF muscle only at a 60° knee extension angle. At the same time, no change occurred at 20° of knee extension (Cavalcante et al., 2021). The VL and RF muscle fibers intermingled on the rectus-vastus aponeurosis attached to the quadriceps tendon (Glenn and Samojla, 2002). Hence, one could suggest that for any knee position placing the RF muscle above its slack length (Xu et al., 2018), an increase in hip extension improves force production capacity of both the RF and the vastii muscles due to the presence of the rectus-vastus aponeurosis and the vastus aponeurosis gathering muscle fibers from all the vastii muscles (Glenn and Samojla, 2002). An apparent mismatch appeared, therefore, between the impairment in maximal voluntary force production capacity that could occur in hip extended position, while intrinsic contractile properties of the knee extensors would be improved.

The maximal force production capacity of the knee extensors can also depend upon neural components driving muscle recruitment. Parallel reduction in voluntary activation and MVIC was reported for the knee extensors when increasing knee extension (Kubo et al., 2004; Doguet et al., 2017b). However, others reported no change in voluntary activation level despite a reduction in MVIC (Becker and Awiszus, 2001; Babault et al., 2003). Less is known about the influence of hip angle position, since to the best of our knowledge, only Maffiuletti and Lepers documented lower voluntary activation level of the knee extensors in a supine than a seated position (Maffiuletti and Lepers, 2003). The scarce and contradictory findings about the impairment of the neural drive to the muscle as a function of the knee or hip joint angle required further investigation to decipher their respective influence.

Using the twitch interpolated technique or the surface EMG analysis of a single muscle of the knee extensors remains limited to infer changes in the voluntary drive during voluntary contraction. Single-pulse transcranial magnetic stimulation could represent an alternative technique to quantify the corticospinal pathway’s excitability and infer the impact of hip or knee angle configuration on the muscles’ neural drive (Weavil and Amann, 2018). A reduction in corticospinal excitability of the VL muscle occurred, for instance, during MVIC of the knee extensors performed at 100° compared to 75° of knee flexion (Doguet et al., 2017a). It has been suggested that joint position may influence corticospinal excitability due to increased Ia afferent discharge affecting spinal excitability at long muscle length (Doguet et al., 2017a) or impairment of neuromechanical properties requiring different neural control strategies (Forman et al., 2016). However, in the absence of muscle length measurement in the study conducted by Doguet et al. (2017a), the extent to which knee angle affects corticospinal excitability through a change in muscle length remains speculative. Furthermore, the influence of hip angulation on central factors driving muscle recruitment during low-intensity voluntary contraction remains to be precise.

In this context, the present study aimed to examine the influence of hip and knee angle on the force production capacity of the knee extensors and determine the neuromuscular and mechanical consequences affecting the VL mono- and RF bi-articular synergistic muscles. We first evaluated maximal torque production capacity and muscle architecture with different knee and hip angle configurations. Secondly, we examined whether knee or hip joint angle influences neural drive of the VL and RF muscles during sub-maximal contractions. We hypothesized that (i) maximal force production capacity of the knee extensors would increase in knee flexed position while the increase in hip flexion would only have a minor effect and (ii) flexed knee angle or hip extended angle would alter recruitment strategies of synergist and antagonists muscles during sub-maximal contraction.

## Materials and Methods

### Participants

Sixteen healthy men with no history of neurological disease and no recent lower limb injury participated in this study (age: 25 ± 7 years; mass: 80.0 ± 6.2 kg; and height: 177 ± 4 cm). Participants were accustomed to experimental protocol investigating neuromuscular function using isokinetic ergometers, percutaneous electrical, and transcranial magnetic stimulations. All participants gave their written informed consent before the experiment. All procedures conformed to the World Medical Association Declaration of Helsinki (2013) and were approved by the French Ethics Committee (ClinicalTrials.gov Identifier: NCT03334526).

### Study Design

**Figure 1:**
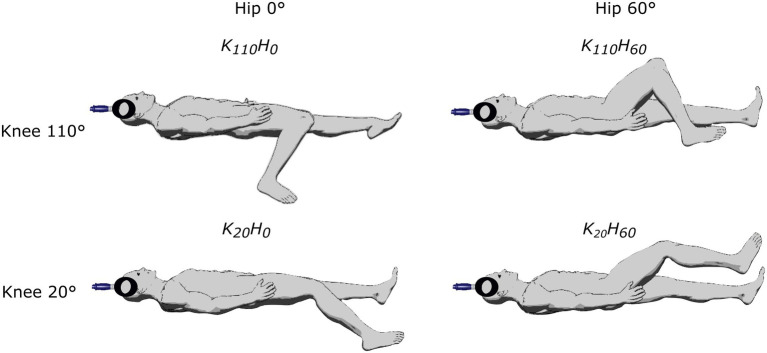
Description of the different positions tested in the experimental protocol by adjusting knee flexion (20° or 110°) and/or hip flexion (0° or 60°).

Participants attended the laboratory twice. The first visit was devoted to neuromuscular testing and the second to the ultrasound measurements. In both sessions, four supine positions were randomly tested on the subject’s dominant leg determined as the kicking leg (one subject was left-legged) using knee angle of either 20° or 110° of flexion (0° = fully extended), and hip angle of either 0° or 60° (0° = fully extended; see Figure 1). Only supine positions were used to avoid the confounding effect of different descending vestibulospinal inputs known to modulate motoneuron excitability (Kennedy et al., 2004). The study was conducted using an isokinetic dynamometer (System Pro 4, Biodex Medical System, New York), and device settings were the same in the two sessions. Participants were lying on their back, the trunk attached with a strap crossing the chest; the axis of the dynamometer was aligned with the knee joint, and the lever arm was attached 2 cm above the malleoli using a non-compliant strap. The thigh was supported by the seat in positions K_110_H_0_ and K_20_H_0_ or using a manufacturer’s device, placed approximately 3 cm upward from the popliteal fossa (positions K_110_H_60_ and K_20_H_60_). The head was aligned in a neutral position and kept fixed by one experimenter throughout the session.

### Neuromuscular Function

For each position, participants performed a warm-up including 10 ramping isometric contractions (60–100% of the subjects’ perceived maximal voluntary contraction). They then performed two 5-s MVIC, with an additional one if the second was 5% greater than the first. Verbal encouragements and visual feedback were provided to the participants during each trial. After that, transcutaneous electrical stimulations were carried out on the femoral nerve using a high-voltage constant-current stimulator (model DS7, Digitimer, Hertfordshire, United Kingdom). A monopolar cathode ball (0.5 cm diameter) was pressed into the femoral triangle, and the anode (10 × 5 cm rectangular electrode) was placed on the gluteal fold opposite the cathode. Single pulses (200-μs width) were used at rest to assess neuromuscular function and during a 20% MVIC contraction for motor evoked potential (MEP) normalization. The optimal stimulation site was determined for each position as the location that evoked the greatest peak twitch and M-wave amplitudes with the same intensity. Once located, stimulation intensity was gradually increased until peak twitch amplitude and M-wave amplitude plateaued and then increased by 20% to ensure supramaximal intensity. Two single pulses were recorded to determine maximal M-wave amplitude. The ratio between MVIC peak torque and amplitude of the single peak twitch (MVIC/Pt) was used to assess the influence of peripheral factors on torque production capacity (Folland et al., 2014).

### Electromyography Recordings

EMG activity of the vastus lateralis and rectus femoris muscles was recorded at a sampling rate of 2 kHz and filtered (10–500 Hz) using Acq-Knowledge analysis software (Model MP150, Biopac System, Santa Barbara, CA) using pairs of pre-gelled Ag/AgCl surface electrodes (recording diameter of 10 mm; Mini KR, Controle Graphique S.A., Brie-Comte-Robert, France). The skin was shaved, abraded, and cleaned with isopropyl alcohol; then, electrodes were taped lengthwise over the middle of the muscle belly with an inter-electrode distance of 20 mm following the SENIAM recommendations (Hermens et al., 2000). The reference electrode was positioned on the contralateral patella. The root-mean square (RMS) value of the EMG was calculated for each muscle over a 100 ms period at the peak torque (i.e., 50 ms before and 50 ms after the peak) during the MVIC and normalized by the corresponding M-wave amplitude (RMS_MVIC_/M). The RMS-EMG of the RF and VL muscles was also calculated over a 100 ms period before the transcranial magnetic stimulation (TMS) stimulus artifact and normalized to the RMS_MVIC_ to control muscle activity during MEP recordings (RMS_MEP_/RMS_MVIC_).

### Corticospinal Excitability

Transcranial magnetic stimulation was delivered with a double-cone coil (110 mm diameter) using a Magstim 200^2^ magnetic stimulator (Magstim, Whitland, Dyfed, United Kingdom) during brief (~3 s) weak knee extensor contractions (20% MVIC of the corresponding position). For each position, the optimal coil position was defined as the position that elicited the greatest MEP amplitude in the VL and the RF muscles with the same stimulus intensity (50% maximal stimulator output) and was marked on the scalp to ensure a constant location. The active motor threshold (AMT) was defined as the lowest stimulation intensity that elicited at least 4 over 8 MEP with a distinguishable silent period from background EMG for both muscles (Sidhu et al., 2013). Input–output curves were constructed between 90 and 170% of the AMT with incremental steps of 10% to obtain maximal MEP amplitude on the VL and RF muscles. Four single pulses per intensity were applied. Maximal MEP peak-to-peak amplitude was analyzed off-line and normalized to the maximal M-wave amplitude of the corresponding position.

### Ultrasound Recordings

**Figure 2:**
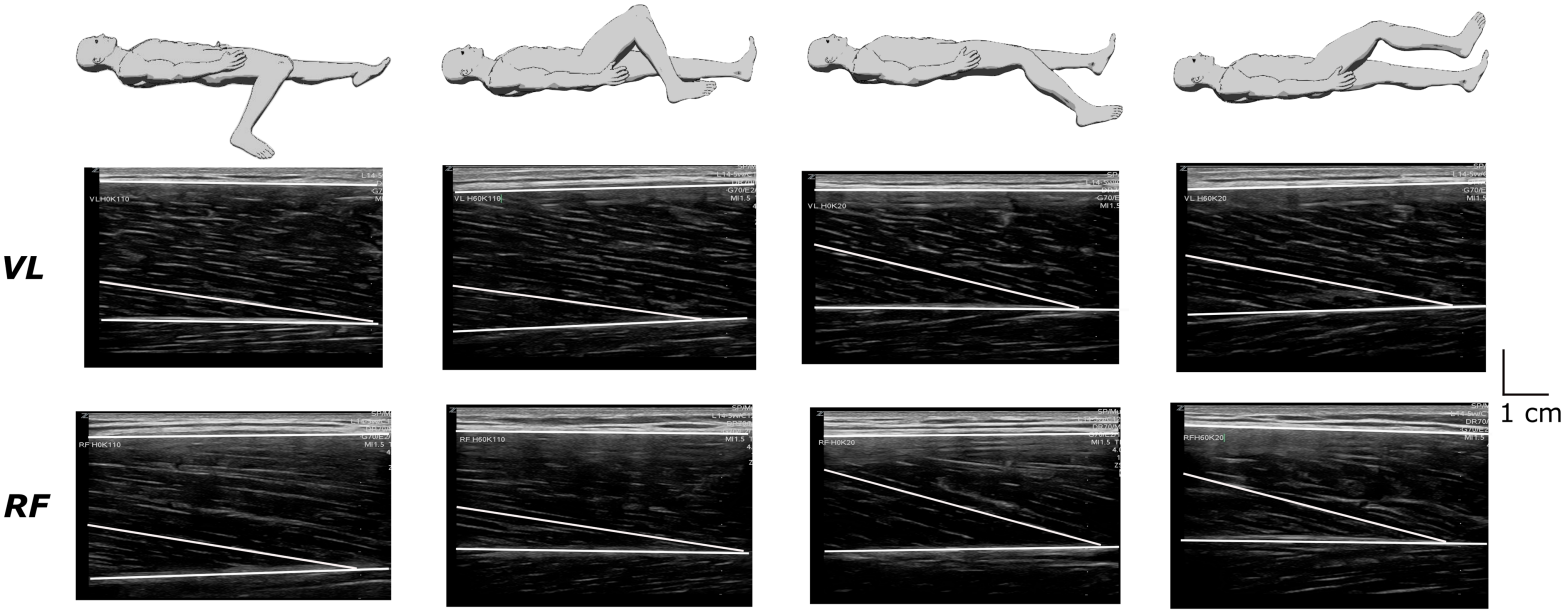
Recordings of ultrasound images from the vastus lateralis (VL) and the rectus femoris (RF) muscles under the different positions in a representative subject.

Ultrasound recordings were performed at rest for the VL and RF muscles using B-mode Zonare ultrasound video imaging (Z. One, Zonare Medical Systems Inc., Mountain View, CA, United States). Determination of muscle architecture at rest was shown to provide a reliable measure of muscle function than when performed during maximal voluntary contraction (Massey et al., 2015). A 5.5-cm (7.5 MHz) linear array probe was positioned perpendicular to the dermal surface and oriented along the longitudinal axis of the muscle-tendon unit. Figure 2 depicts examples of ultrasound recordings for a representative subject. Images were collected at 50% of muscle length (M_L_) to limit fascicles and aponeurosis curvature and favor a relatively isotropic muscular architecture (Blazevich et al., 2006). Muscle length was calculated as the distance between the proximal and the distal myotendinous junction, determined from the convergence of the deep and superficial aponeuroses. Once identified and localized with the probe, the position of the myotendinous junctions was marked on the skin to allow muscle length measurement. Three images were stored for each muscle to calculate off-line fascicle length (*F_L_*) and pennation angle (*Pα*) using Kinovea© (0.8.15 2006–2011; Joan Charmant & Contrib, Bordeaux, France). Criteria for storing images were as: parallel superficial and deep aponeurosis and the presence of at least three discernible fascicles with their junction on the deep aponeurosis to determine *Pα*. Two to three fascicles were analyzed on each image to calculate *F_L_* using the extrapolation method validated *in vivo* by Brennan et al. (2017), where *h* is the distance between the intersection point of the visible fascicle with the edge of the image and the superficial aponeurosis:


$$F_{L}=\mathrm{visible fascicle length}+\frac{h}{\sin\left(P\alpha \right)}$$

### Statistical Analysis

All data are presented as mean ± standard deviation (SD) in text, tables, and figures. The nature of the distribution was assessed for all variables using the Shapiro–Wilk test. Data of maximal MEP amplitude of the RF muscle were log-transformed to ensure the relevant use of parametric testing since the data were not normally distributed. Then, sphericity was checked as appropriate, and a Greenhouse–Geisser correction to the degree of freedom was applied when sphericity was violated. One-way ANOVA tested the effect of *position*, and when significant, the main effect was followed up with a Tukey HSD test. Effect sizes are reported as partial eta squared (*η_p_*^2^). Statistical analyses were performed with Statistica (StatSoft France, version 7.1, STATISTICA), and the Cohen’s *d_z_* effect size was calculated using G*Power 3.1 (Faul et al., 2007). The significance level was set at 0.05 (two tailed) for all analyses.

## Results

### Neuromuscular Function

**Figure 3:**
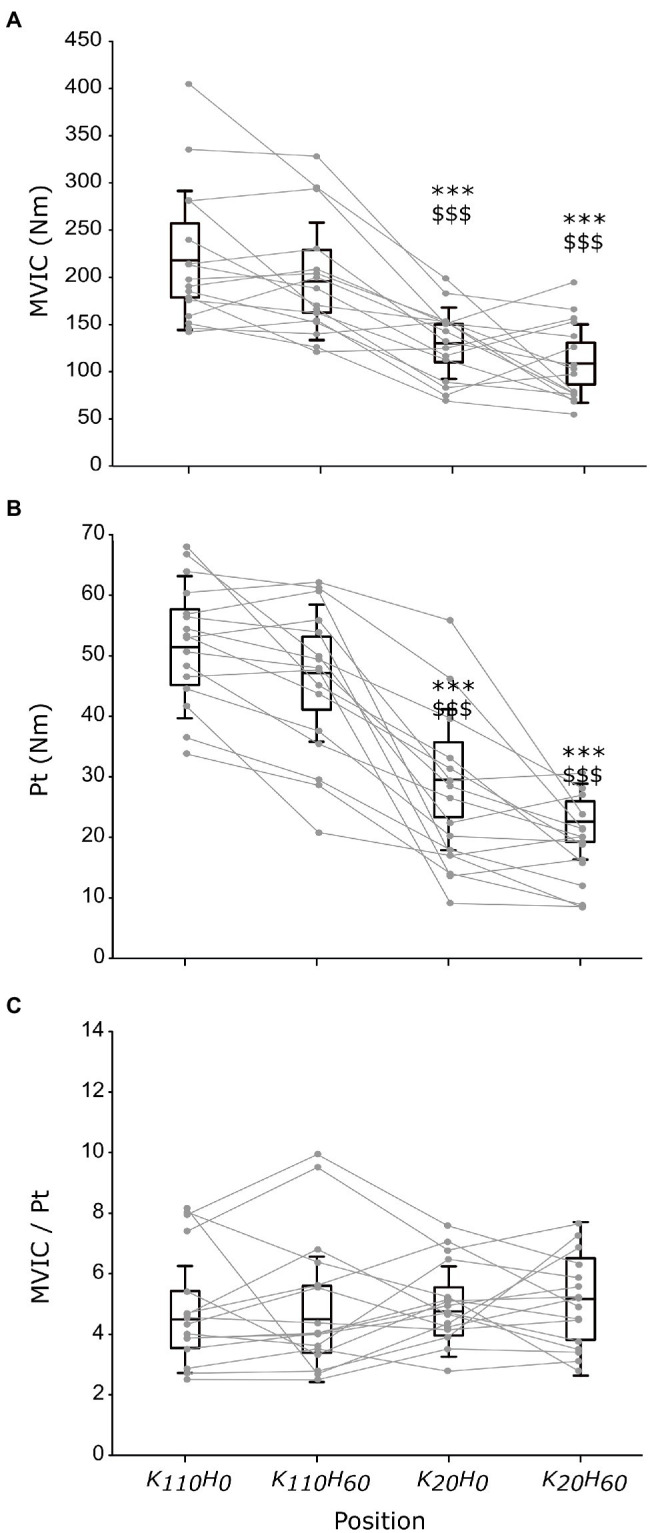
Maximal torque production capacity of the knee extensors measured in each position during maximal voluntary isometric contraction (MVIC; **A**) or elicited by percutaneous nerve stimulation **(B)**, and the ratio between MVIC and peak twitch maximal responses (**C**; *n* = 16). Black boxes and whisker plots represent the average values for all participants (line: mean, box: 95% CI, and whiskers: SD), individual data are provided in grey. ^*^ or ^$^ denotes significant difference from position K_110_H_0_ or K_110_H_60_, respectively (*p* < 0.001).

The ANOVA detected a main effect of *position* on MVIC (*p* < 0.001; *η_p_*^2^ = 0.648; see Figure 3A) and Pt (*p* < 0.001; *η_p_*^2^ = 0.762; see Figure 3B). Both parameters were greater for positions K_110_H_0_ and K_110_H_60_ than positions K_20_H_0_ and K_20_H_60_ (all *p* < 0.001; all *d_z_* > 1.184). No difference occurred between K_110_H_0_ and K_110_H_60_ or K_20_H_0_ and K_20_H_60_ (all *p* > 0.081). No difference was detected for the MVIC/Pt ratio (*p* = 0.528; *η_p_*^2^ = 0.048; see Figure 3C). The ANOVA revealed no difference in stimulation intensity applied to evoke maximal M-wave amplitude between positions (mean across all positions 199.8 ± 27.0 mA; *p* = 0.447; *η_p_*^2^ = 0.048). The ANOVA detected no *position* effect on M-wave amplitude for both the VL (*p* = 0.153; *η_p_*^2^ = 0.109) and the RF muscles (*p* = 0.192; *η_p_*^2^ = 0.099; see Table 1). No main effect was detected for the RMS_MVIC_/M ratio for both the VL (*p* = 0.265; *η_p_*^2^ = 0.089) and the RF muscles (*p* = 0.186; *η_p_*^2^ = 0.115; see Table 1).

**Table 1:** Electrophysiological parameters recorded for the vastus lateralis and rectus femoris muscles in the four positions (*n* = 16; mean ± SD).

	Positions			
	K_110_H_0_	K_110_H_60_	K_20_H_0_	K_20_H_60_
**M-wave amplitude (mV)**				
VL	7.38 ± 3.1	6.61 ± 3.64	8.45 ± 4.55	7.7 ± 5.13
RF	2.91 ± 1.34	2.84 ± 1.73	3.56 ± 2.06	3.34 ± 1.77
**RMS_MVIC_/M (×10^−2^)**				
VL	8.74 ± 4.29	9.66 ± 6.34	7.94 ± 4.38	10.6 ± 5.19
RF	8.41 ± 4.74	12.82 ± 8.87	10.17 ± 6.62	12.52 ± 8.42
**MEP amplitude (mV.Mmax)**				
VL	0.32 ± 0.10	0.38 ± 0.20	0.31 ± 0.14	0.23 ± 0.11^$^
RF	0.42 ± 0.27	0.61 ± 0.38	0.39 ± 0.27	0.43 ± 0.31
RMS_MEP_/RMS_MVIC_				
VL	0.13 ± 0.08	0.14 ± 0.08	0.13 ± 0.10	0.09 ± 0.07^$^
RF	0.22 ± 0.10^$^	0.33 ± 0.15	0.28 ± 0.18	0.25 ± 0.27^$^

RMS_MVIC_/M, ratio of the root-mean square of the raw EMG recorded during maximal voluntary contraction and the maximal M-wave peak-to-peak amplitude; RMS_MEP_/RMS_MVIC_, ratio of the root-mean square of the raw EMG recorded during 20% MVIC contraction before the MEP and EMG recorded during MVIC.

$ Significantly different from position K_110_H_60_ (*p* < 0.05).

### Muscle Architecture

**Figure 4:**
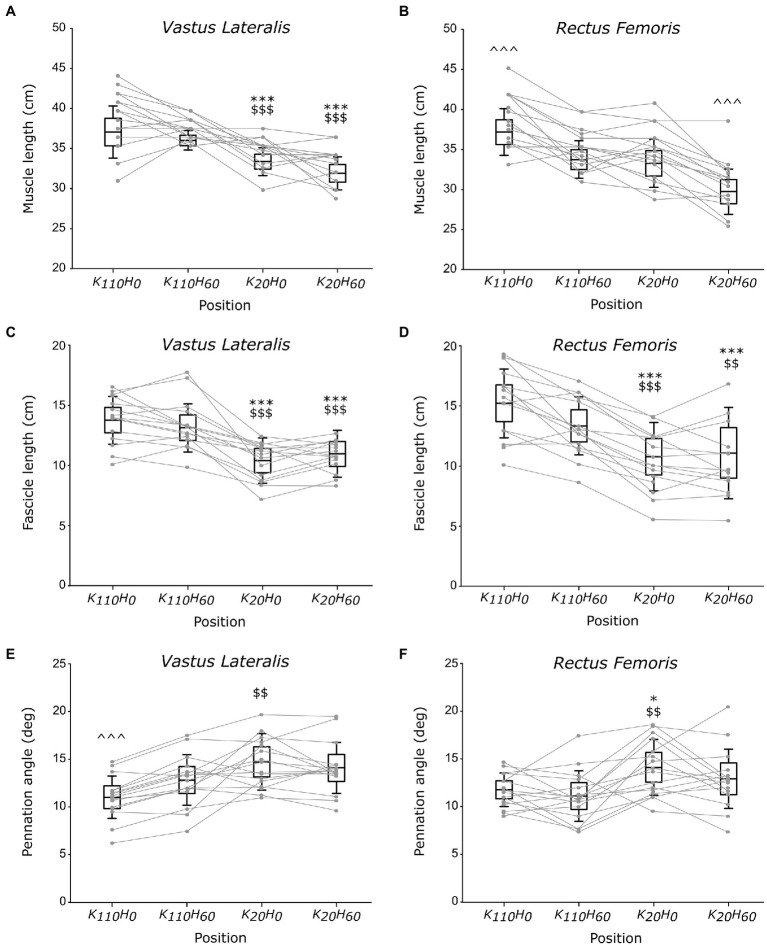
Architectural parameters of the vastus lateralis (VL) and rectus femoris (RF) muscles; muscle **(A,B)** and fascicle **(C,D)** length and pennation angle (**E,F**; *n* = 16). Black boxes and whisker plots represent the average values for all participants (line: mean, box: 95% CI, and whiskers: SD), individual data are provided in grey. For each muscle, ^*^ or ^$^ or ^ denotes significant difference from position K_110_H_0_ or K_110_H_60_ or all other positions, respectively. One, two, or three symbols indicate difference at *p* < 0.05, *p* < 0.01, or *p* < 0.001, respectively.

A main position effect was detected on muscle length for the VL and RF muscles (all *p* < 0.001; all *η_p_*^2^ > 0.637; see Figures 4A, B). VL muscle length was greater in positions K_110_H_0_ and K_110_H_60_ compared to positions K_20_H_0_ and K_20_H_60_ (all *p* < 0.001; all *d_z_* > 1.134), without difference between positions K_110_H_0_ and K_110_H_60_ or K_20_H_0_ and K_20_H_60_ (all *p* > 0.126). The RF muscle was significantly longer in position K_110_H_0_ (all *p* < 0.001; all *d_z_* > 1.335) and shorter in position K_20_H_60_ (all *p* < 0.00; all *d_z_* > 1.518) compared to all other positions. No difference in RF muscle length was detected between positions K_110_H_60_ and K_20_H_0_ (*p* = 0.843). Significant differences in fascicle length also occurred between positions for both the VL and RF muscles (all *p* < 0.001; all *η_p_*^2^ > 0.542; see Figures 4C,D). Both muscles demonstrated greater *F_L_* in positions K_110_H_0_ and K_110_H_60_ than K_20_H_0_ and K_20_H_60_ (all *p* < 0.017; all *d_z_* > 0.693). No difference between positions K_110_H_0_ and K_110_H_60_ (all *p* > 0.094) or K_20_H_0_ and K_20_H_60_ (all *p* > 0.578) was detected for *F_L_* of the VL and RF muscles. A significant *position* effect was also evidenced on *Pα* for the two muscles (all *p* < 0.003; all *η_p_*^2^ > 0.269; see Figures 4E,F). The VL muscle demonstrated significantly lower *Pα* in position K_110_H_0_ than all other positions (all *p* < 0.010; all *d_z_* > 0.353). *Pα* of the VL muscle was also lower in position K_110_H_60_ than K_20_H_0_ (*p* = 0.006; *d_z_* = 0.707), but no difference was detected between position K_20_H_60_ and K_20_H_0_ or K_110_H_60_ (all *p* > 0.102). The RF muscle demonstrated significantly greater *Pα* in position K_20_H_0_ than K_110_H_0_ and K_110_H_60_ (all *p* < 0.025; all *d_z_* > 1.019). No other difference was reported on *Pα* for the RF muscle.

### Corticospinal Properties

No difference was reported between positions in stimulator intensity used to assess the AMT (mean across all positions 33 ± 4% maximal stimulator output; *p* = 0.739; *η_p_*^2^ = 0.027) or to elicit maximal MEP amplitude (mean across all positions 158 ± 0.91% AMT; *p* = 0.522, *η_p_*^2^ = 0.048). The ANOVA detected a main *position* effect on the RMS_MEP_/RMS_MVIC_ ratio for the VL and RF muscles (all *p* < 0.015; all *η_p_*^2^ > 0.218; see Table 1). The ratio was higher for the VL muscle in position K_110_H_60_ than K_20_H_60_ (*p* = 0.014; *d_z_* = 0.241). The RMS_MEP_/RMS_MVIC_ ratio of the RF muscle was higher in position K_110_H_60_ compared to positions K_20_H_60_ and K_110_H_0_ (all *p* < 0.024; all *d_z_* > 0.296). The ANOVA detected a significant *position* effect on maximal MEP amplitude for the VL muscle (*p* = 0.029; *η_p_*^2^ = 0.180; see Table 1) being greater in position K_110_H_60_ than K_20_H_60_ (*p* = 0.017; *d_z_* = 0.712). No main effect was detected on maximal MEP amplitude of the RF muscle (*p* = 0.157; *η_p_*^2^ = 0.108; see Table 1).

## Discussion

This study sought to examine the influence of hip and knee angle position on maximal force production capacity and their consequence on muscle architecture and neural drive of knee extensors. The main findings validated our first hypothesis, whereby knee flexion increased maximal force production capacity of the knee extensors highlighted by greater voluntary and electrically evoked contraction torques, while the hip position does not influence maximal force production capacity. However, our findings invalidate our second hypothesis about alteration in the neural drive of the knee extensors in knee extended or hip flexed position since no change in muscle activity or corticospinal excitability occurred between the different positions. The knee flexed positions also resulted in significant changes in muscle architecture of the VL and RF muscles, with greater fascicule length and lower pennation angle. These elements suggest a non-negligible influence of the knee extensors’ contractile properties on the torque production capacity of the knee extensors rather than neural components.

The greater MVIC torque of the knee extensors recorded in knee flexed (i.e., 110° flexion) compared to knee extended position (i.e., 20° flexion) highlights the prominent effect that knee angle position exerts on MVIC of the knee extensors (Babault et al., 2003; Cavalcante et al., 2021). The absence of difference between MVIC achieved in a 0° or 60° hip flexion position irrespective of knee angle position confirmed that hip angle position does not influence knee extensors maximal force production capacity (Bampouras et al., 2017; Cavalcante et al., 2021). However, these findings contradict the reduction in MVIC reported in hip extended position than hip flexed position (Maffiuletti and Lepers, 2003; Rochette et al., 2003; Ema et al., 2017). The constant EMG RMS_MVIC_/M ratio of the VL and RF muscles between positions suggests that reduction in MVIC does not result from altered muscle recruitment (Becker and Awiszus, 2001; Babault et al., 2003). A difference in maximal MEP amplitude was only reported for the VL muscle with greater amplitude in position K_110_H_60_ than K_20_H_60_. This increase in corticospinal activity could be explained by a greater muscle activity transcribed by the higher RMS_MEP_/RMS_MVIC_ ratio observed both for the VL and RF muscle, suggesting that neural drive during voluntary contraction of low intensity adapts likely to overcome a deficit in mechanical disadvantages (Babault et al., 2003). Neither the intensity required to evoke maximal twitch torque amplitude nor the amplitude of the maximal M-wave was different between positions. However, the evoked twitch torque amplitude was impaired similarly to MVIC by the knee angle position (i.e., greater at 110° than 20° knee flexion) while unaffected by the hip angle position. Taken together, these findings suggest that reduction in MVIC occurring in the knee extended positions resulted from impairments in the excitation-contraction coupling process. Strengthen by the constant MVIC/Pt ratio between positions (see Figure 3C), our findings suggest that contractile properties were a determinant factor in force production capacity of the knee extensors in the investigated positions (Folland et al., 2014). Because of the change in knee position angle, impairments in contractile properties may partly result from a difference in muscle length and architecture conditioning their force production capacity (Gordon et al., 1966; Eng et al., 2018).

The use of ultrasonography in the present study was intended to characterize the influence of hip and knee angle positions on muscular architecture and infer their impact on contractile properties of the knee extensors. The architectural properties of the VL and RF muscles (i.e., fascicle length and pennation angle) reported in the present study are consistent with other measurements made in previous studies (Blazevich et al., 2003; Cavalcante et al., 2021). Despite a direct link between the RF and the VL muscle ensured by the presence of the rectus-vastus aponeurosis (Glenn and Samojla, 2002), current findings demonstrated that VL muscle length varied only as a function of knee angle, being significantly greater in knee flexed position than knee extended position irrespectively of hip angle position. Significant differences in RF muscle length occurred when hip and knee angles varied in the opposite direction; significantly shorter or longer muscle length was measured in positions K_20_H_60_ or K_110_H_0_ than all other positions, respectively. Changes in fascicle length followed a similar trend for the two investigated muscles, being greater in positions K_110_H_0_ and K_110_H_60_ than in positions K_20_H_0_ and K_20_H_60_. Therefore, these findings suggest that only knee angle position affected the fascicle length of the VL and RF muscle while hip angle position did not. One should note that the higher MVIC observed in the present study was developed in positions that significantly increase the fascicle length of the VL and RF muscles. In accordance with the sliding filament theory (Gordon et al., 1966), the present findings suggest that the greater MVIC developed in the more flexed knee positions would transcribe a more favorable fascicle length, increasing the number of actin-myosin bridges that overlap during contraction.

Another interesting finding of the present study concerns changes in pennation angle across the different positions. Indeed, our results showed lower Pα of the VL muscle in position K_110_H_0_ than K_20_H_0_, while no difference occurred between position K_20_H_60_ and K_110_H_60_. Additionally, lower Pα of the VL muscle was observed in position K_110_H_0_ than K_110_H_60_, while no difference occurred between position K_20_H_0_ and K_20_H_60_. Taken together, these findings showed that a knee flexed position (i.e., 110°) decreases Pα of the VL muscle compared to a knee extended position (i.e., 20°) only when the hip joint was in an extended position (i.e., 0°), but not when placed in a flexed position (i.e., 60°). Alternatively, the hip extension can also decrease Pα of the VL muscle when the knee joint angle is flexed but not when positioned in an extended position. Present findings also demonstrated lower Pα of the RF muscle in position K_110_H_0_ than position K_20_H_0_, while no difference was observed between positions K_110_H_0_ and K_110_H_60_ or positions K_20_H_0_ and K_20_H_60_. These findings also showed that Pα of the RF muscle was decreased only in response to an increase in knee flexion when the hip is extended, but not when the hip is in a flexed position, and that change in the hip angle position has no influence on *Pα* of the RF muscle irrespectively of the knee position. These findings demonstrate thus that an increase in hip extension can decrease *Pα* of the VL muscle only when the knee was flexed at 110°. In contrast, a decrease in *Pα* of the RF muscle occurred when VL muscle length increased and the hip joint was extended, while no change in *Pα* occurred for the RF muscle when VL muscle remained constant. Altogether, these findings demonstrate that the VL and RF muscles can mutually influence on *Pα* of their synergist counterpart, particularly when the hip was extended or the knee was flexed. One could suggest that this mutual influence that each muscle exerted on its synergist counterpart can partly be explained by the strong bonds that their respective muscle fibers form when attaching together into the rectus-vastus aponeurosis (Glenn and Samojla, 2002). Therefore, these findings agreed with the conclusion drawn by Glenn and Samojla (2002), whereby the VL and RF muscle would improve force production when they function similarly to a bipennate muscle thanks to their structural link provided by the rectus-vastus aponeurosis. Furthermore, it appeared that the influence that the VL and RF muscles exert on *Pα* of their counterpart would make it possible only for knee joint angle placing these muscles beyond their slack angle (Xu et al., 2018). However, it remains to determine whether this finding also holds for the hip joint angle by determining its slack angle.

### Limitations and Recommendations for Future Studies

Despite consistent findings arguing in favor of the predominance of contractile properties over the neural drive into the force production capacity of the knee extensors, this study is not exempt from limitations that should be taken into account in future studies. The architecture of the RF muscle could characterize that of the VM or VL muscles, while the VL muscle appears less suitable to infer the architectural characteristics of the other constituent of the knee extensors (Blazevich et al., 2006). Xu et al. (2018) reported that the stiffness of the VMO and the VL muscles increases to the same extent in response to knee flexion, which could suggest similar changes in the architecture between these two muscles. However, the absence of ultrasonography measurements from the VM muscle in the present study avoids any conclusions about the behavior of the VM muscle in response to the knee or hip angle changes and requires specific architectural investigations to precise its sensitivity to joint angle changes. Furthermore, the within-muscle heterogeneity of architectural characteristics of the RF and VL muscles highlighted by Blazevich et al. (2006) makes impossible to conclude that changes occurring in the middle site in the present study also occurred at the proximal or distal sites. Further studies should therefore investigate different sites along each muscle to describe precisely whether proximal and distal portions of the knee extensors present similar architectural changes to those highlighted at the medial portion in the present study. The use of surface EMG also presents some limitations to infer the central drive sent to the knee extensors, since different recruitment strategies were highlighted between the VL and VM muscles during voluntary contraction. Specifically, the VM muscle seems less activated than the VL muscle under knee extended position (Visscher et al., 2017), which prevents us from generalizing the behavior of the VL muscle to the VM muscle. Further studies should therefore complete the neural drive sent to the VM muscle during voluntary contractions under different knee and hip angles configurations, using surface electromyography recordings as well as the interpolated twitch procedure to add clues about the voluntary activation level of the knee extensors under different configurations.

## Conclusion

The present results suggest that reduction in MVIC, reported for knee extended positions, was mainly due to mechanical disadvantage, particularly short fascicle length leading to an inadequate actin-myosin overlap. In accordance with a recent study (Cavalcante et al., 2021), the different hip angulations tested in the present study did not impair the force production capacity of the knee extensors. This discrepancy with precedent findings (Maffiuletti and Lepers, 2003; Rochette et al., 2003; Ema et al., 2017) might be explained by the different range of simultaneous change in hip and knee angle used in the present study compared to previous ones. Our findings also showed that the hip angle position could influence the change in pennation angle of the VL muscle, which suggests that the RF muscle state can exert a non-negligible influence on VL architecture. Future studies should be conducted to determine more precisely the mutual influence that the VL and the RF muscle can exert on each other through the rectus-vastus aponeurosis and the behavior of the two other heads of the knee extensors (vastus medialis and vastus intermedius).

## Data Availability Statement

The raw data supporting the conclusions of this article will be made available by the authors, without undue reservation.

## Ethics Statement

The studies involving human participants were reviewed and approved by the French Ethics Committee (ClinicalTrials.gov Identifier: NCT03334526). The patients/participants provided their written informed consent to participate in this study.

## Author Contributions

YG, RL, AM, and CP conceived and designed the work. All authors acquired, analyzed or interpreted the data of the work, and drafted and revised critically the work. All authors approved the final version of the manuscript. All authors agree to be accountable for all aspects of the work in ensuring that questions related to the accuracy or integrity of any part of the work are appropriately investigated and resolved. All persons designated as authors qualify for authorship, and all those who qualify for authorship are listed.

## Conflict of Interest

The authors declare that the research was conducted in the absence of any commercial or financial relationships that could be construed as a potential conflict of interest.

## Publisher’s Note

All claims expressed in this article are solely those of the authors and do not necessarily represent those of their affiliated organizations, or those of the publisher, the editors and the reviewers. Any product that may be evaluated in this article, or claim that may be made by its manufacturer, is not guaranteed or endorsed by the publisher.
